# Abraxane-induced bone marrow CD11b^+^ myeloid cell depletion in tumor-bearing mice is visualized by μPET-CT with ^64^Cu-labeled anti-CD11b and prevented by anti-CSF-1

**DOI:** 10.7150/thno.49421

**Published:** 2021-01-20

**Authors:** Qizhen Cao, Qian Huang, Y. Alan Wang, Chun Li

**Affiliations:** 1 Department of Cancer Systems Imaging, The University of Texas MD Anderson Cancer Center, Houston, TX 77054; 2 Department of Cancer Biology, The University of Texas MD Anderson Cancer Center, Houston, TX 77054

**Keywords:** anti-CD11b, μPET-CT imaging, anti-CSF-1, Abraxane, bone marrow toxicity

## Abstract

To investigate the utility of noninvasive µPET-CT with ^64^Cu-DOTA-anti-CD11b (^64^Cu-αCD11b) in assessing bone marrow status after anticancer therapies, and the protective role of anti-CSF-1 (αCSF-1) against bone marrow suppression induced by Abraxane.

**Methods:** MDA-MB-435 tumor-bearing mice were treated with Abraxane, αCSF-1, or αCSF-1 plus Abraxane. µPET-CT and biodistribution of ^64^Cu-αCD11b were performed after intravenous injection of the radiotracer. Cells from mouse bone marrow and MDA-MB-435 tumor were analyzed by flow cytometry. A humanized αCSF-1 was investigated for its role in protecting bone marrow cells, using a transgenic mouse model that expresses functional human CSF-1.

**Results:** μPET-CT showed that ^64^Cu-αCD11b had high uptake in the bone marrow and spleen of both normal and tumor-bearing mice. Abraxane significantly reduced ^64^Cu-αCD11b uptake in the bone marrow and spleen of treated mice compared to untreated mice. Interestingly, ^64^Cu-αCD11b μPET-CT revealed that αCSF-1 alleviated the depletion of bone marrow cells by Abraxane. These changes in the bone marrow population of CD11b^+^ myeloid cells were confirmed by flow cytometry. Moreover, αCSF-1 potently enhanced tolerance of bone marrow granulocytic myeloid cells to Abraxane, decreased cell migration, and suppressed recruitment of myeloid cells to the tumor microenvironment. The humanized αCSF-1 also alleviated the effects of Abraxane on bone marrow cells in transgenic mice expressing human CSF-1, suggesting clinical relevance of αCSF-1 in prevention of bone marrow suppression in addition to its role in reducing tumor-infiltrating myeloid cells.

**Conclusions:** Abraxane-induced bone marrow CD11b^+^ myeloid cell depletion in tumor-bearing mice could be noninvasively assessed by μPET-CT with ^64^Cu-αCD11b and prevented by αCSF-1.

## Introduction

There is growing interest in combining chemotherapy with immunotherapy. In tumor infiltrates, macrophages and myeloid-derived suppressor cells (MDSCs) are often the most abundant immune cells and play a critical role in promoting tumor growth. Macrophage colony-stimulating factor (M-CSF; also known as CSF-1) is a potent growth factor that promotes the differentiation, proliferation, and migration of monocytes and macrophages via signaling through its receptor tyrosine kinase CSF-1R. In preclinical models, blockade of CSF-1/CSF-1R signaling inhibited recruitment of bone marrow myeloid cells to the tumor during chemotherapy and induced type I interferon response, thereby reversing chemoresistance and sensitizing tumor cells to chemotherapy 1-3. For example, in preclinical breast cancer models, combining CSF-1/CSF-1R blockade, which depletes tumor-associated macrophages and MDSCs, with paclitaxel or cisplatin chemotherapy has shown increased antitumor efficacy 3, 4. However, the impact of CSF-1/CSF-1R blockade and its combination with chemotherapy on the myeloid cells in bone marrow and spleen is poorly defined. For clinical studies, analyzing the effects of such combination therapy on the cellular compartment of the bone and spleen either involves invasive procedures, which subjects to sampling error (bone marrow aspiration) or is not practical (*i.e.*, removing spleen tissue for analysis). The hematopoietic component of bone marrow produces approximately 500 billion new blood cells per day in order to maintain steady-state levels in the peripheral circulation 5. Because macrophages and MDSCs in the tumors are mostly recruited from the bone barrow and spleen, we hypothesized that changes in myeloid cells during combination therapies between chemotherapy and immunotherapy could be noninvasively assessed by positron emission tomography-computed tomography (PET-CT). The ability to visualize myeloid cell trafficking throughout the body would deepen our understanding of the mechanisms of drugs' action, which would facilitate the rational design of combination strategies, including defining injection dose and dosing interval of combination therapy.

CD11b is expressed on the surface of myeloid cells, including monocytes, granulocytes, macrophages, natural killer cells, and dendritic cells. CD11b regulates myeloid cell adhesion and migration and has been implicated in several immune processes, including phagocytosis, cell-mediated cytotoxicity, chemotaxis, and activation of signal transduction 6. Bone marrow contains hemopoietic stem cells, progenitor cells that differentiate into CD11b-positive (CD11b^+^) myeloid cells. We previously showed that ^64^Cu-labeled anti-CD11b (^64^Cu-αCD11b) specifically binds to CD11b^+^ myeloid cells *in vitro* and *in vivo* in immunocompetent mice 7. Based on these data, we further hypothesized that μPET-CT with probes directed at CD11b^+^ could be used for successful assessment of chemotherapy-induced depletion of bone marrow cells and prevention of chemotherapy-induced depletion of bone marrow cells by anti-CSF-1 (αCSF-1).

We tested our hypotheses in MDA-MB-435 tumor-bearing nude mice and human CSF-1 knock-in mice treated with albumin-bound paclitaxel (Abraxane). We found that µPET-CT imaging with ^64^Cu-labeled rat anti-mouse CD11b could visualize changes of bone marrow cell density in response to therapy with Abraxane. In addition, we found that αCSF-1 alleviated Abraxane-mediated depletion of CD11b^+^ cells in the bone marrow. Our findings were further confirmed using humanized αCSF-1 in a transgenic mouse model that expresses functional human CSF-1. These results suggest that αCSF-1 may have a role in protecting bone marrow from chemotherapy-induced myeloid suppression, and that ^64^Cu-αCD11b μPET-CT could be an alternative to biopsies as a noninvasive assessment of functional bone marrow following therapies.

## Materials and Methods

### Antibodies and other reagents

Rat anti-mouse CD11b (clone M1/70; αCD11b) and its phycoerythrin conjugate, rat anti-mouse CD45 FITC (clone 104), rat anti-mouse CD169 eFluor660 (clone SER-4), rat anti-mouse Ly6C allophycocyanin (clone hk1.4), rat anti-mouse Ly6G (Gr-1) PerCP.Cyanine5.5 (clone RB6-8C5), and rat anti-mouse CD127 APC-eFluor780 (clone A7R34) were purchased from eBioscience, Inc (San Diego, CA). Rat anti-mouse Ly6G FITC was purchased from BD Pharmingen (clone 1A8) (San Jose, CA). p-SCN-Bn-DOTA was purchased from Macrocyclics, Inc (Dallas, TX). Abraxane (10% paclitaxel and 90% human serum albumin) was purchased from Abraxis Oncology (Bridgewater, NJ). Anti-mouse CSF-1 antibody (αCSF-1, AM027, a rat IgG1 anti-mouse CSF-1 antibody, clone number 5A1 8) was obtained from the University of California San Francisco. Anti-human CSF-1 antibody (PD-0360324, a humanized IgG2 monoclonal antibody) was kindly provided by Pfizer Inc. (New York City, NY). ^64^CuCl_2_ was obtained from the Cyclotron Radiochemistry Facility at The University of Texas MD Anderson Cancer Center.

### DOTA conjugation and radiolabeling of αCD11b

p-SCN-Bn-DOTA was added to αCD11b at a molar ratio of 20 : 1 or 50 : 1 in 0.1 M sodium bicarbonate buffer (pH 8.5). The resulting conjugate, DOTA-αCD11b, was purified by PD-10 column and concentrated by Centricon filter (Millipore, Bedford, MA). The final DOTA : αCD11b ratio (number of DOTA per αCD11b) was measured according to reported procedure 9, 10. There were 2.54 ± 0.28 and 5.77 ± 0.39 DOTA moieties per αCD11b in the 20 : 1 and 50 : 1 preparation, respectively (**Table S1**). For radiolabeling, ^64^CuCl_2_ was diluted with 0.2 mL of 0.1 M sodium acetate buffer, and the pH of the solution was adjusted to pH 6.0 with 1 N NaOH. DOTA-αCD11b (~25 µg of DOTA-αCD11b obtained from the 20 : 1 DOTA : αCD11b molar ratio; ~10 µg DOTA-αCD11b obtained from the 50 : 1 DOTA : αCD11b molar ratio) was then added into sodium acetate-buffer solution containing 37 MBq of ^64^CuCl_2_ and incubated for 1 h at 38 °C with constant shaking. The resulting ^64^Cu-αCD11b was purified by PD-10 column using phosphate-buffered saline (PBS) as the mobile phase 10.

### Cell lines

Human MDA-MB-435 melanoma cells were purchased from ATCC (Manassas, VA) and were reconfirmed with short tandem repeat DNA fingerprinting method by the Characterized Cell Line Core Facility of MD Anderson Cancer Center. The MDA-MB-435 cells were maintained in Leibovitz's L-15 medium (Invitrogen) supplemented with 10% fetal bovine serum at 37 ºC.

### Animal models

All animal experiments were performed in compliance with the guidelines established by the MD Anderson Cancer Center Institutional Animal Care and Use Committee. Female nude mice of 6 - 8 weeks of age were obtained from Taconic (Cambridge City, IN) and were kept under sterile conditions. Female JAX C;129S4-*Rag2^tm1.1Flv^* CSF1*^tm1(CSF1)Flv^ Il2rg^tm1.1Flv^*/J mice (stock # 017708; human M-CSF [CSF-1] knock-in) of 6 - 8 weeks of age were purchased from Jackson Laboratory (Bar Harbor, ME) and were housed under sterile conditions. These mice express functional human CSF-1 11.

MDA-MB-435 human cancer cells (5×10^6^) in PBS (100 μL) were inoculated subcutaneously into the right shoulder of female nude mice and JAX CSF-1 knock-in mice. Tumor growth was monitored by digital caliper. When MDA-MB-435 tumors grew to approximately 100 - 150 mm^3^, mice were randomly divided into different groups (n = 3 - 5 mice per group) for treatment with a single dose or multiple doses of Abraxane. Anti-mouse CSF-1 antibody was used in the nude mice model; and anti-human CSF-1 antibody was used in the JAX human CSF-1 knock-in transgenic mice model.

In the Abraxane single-dose regimen, mice were treated as follows: group 1, no treatment (control); group 2, intravenous injection of Abraxane at an equivalent paclitaxel dose of 30 mg/kg on day 2; and group 3, intraperitoneal injection of αCSF-1 on day 0 (1 mg/mouse) and day 4 (0.5 mg/mouse) and intravenous injection of Abraxane (30 mg equivalent paclitaxel/kg) on day 2. In the Abraxane multidose (3 doses) regimen, mice were treated as follows: group 1, no treatment (control); group 2, intraperitoneal injection of αCSF-1 (1 mg/mouse) on days 0, 4, 8, and 12; group 3, intravenous injection of Abraxane at an equivalent paclitaxel does of 30 mg/kg per injection on days 2, 6, and 10; and group 4, intraperitoneal injection of αCSF-1 (1 mg/mouse) on days 0, 4, 8, and 12 plus intravenous injection of Abraxane (equivalent paclitaxel dose of 30 mg/kg/injection) on days 2, 6, and 10.

### µPET-CT imaging

µPET-CT images were acquired using an Inveon scanner (Siemens Medical Solutions, Erlangen, Germany) or an Albira scanner (Bruker, Billerica, MA). In the Abraxane single-dose treatment protocol, each mouse was injected with 7.1 ± 0.28 MBq of ^64^Cu-αCD11b (20 : 1 DOTA to αCD11b molar ratio, 4.8 ± 0.19 µg of αCD11b/mouse) via tail vein on day 4 (2 days after Abraxane injection), and images were acquired 24 h after injection with the Inveon scanner. In the Abraxane multidose treatment protocol, each mouse was injected with 5.1 ± 0.52 MBq of ^64^Cu-αCD11b (50 : 1 DOTA to αCD11b molar ratio, 1.4 ± 0.14 µg of αCD11b/mouse) intravenously on day 12 after the initiation of treatments, and PET images were acquired 24 h after injection with the Albira scanner.

For each µPET scan, 3-dimensional regions of interest were drawn over the major organs on decay-corrected whole-body coronal images. The average radioactivity in each region of interest was obtained to derive percentage of injected dose per gram of tissue (%ID/g) values with Siemens Inveon Research Workplace software or percentage injected dose per cubic centimeter (%ID/cc) values with Bruker-Albira PMOD analysis software, version 4.0.

### Biodistribution studies

Mice from different treatment groups were euthanized 48 h after radiotracer injection at the end of imaging sessions. Blood, major organs, and tumor tissues were collected, wet-weighed, and counted with a γ-counter (Packard, Waltham, MA). The results were normalized to 25-g mouse body weight and reported as mean %ID/g ± standard deviation (n = 3/group).

### Cell counting and flow cytometry

Bone marrow cells were obtained from the femur of female nude mice according to reported procedures 12, 13. Briefly, cells from bone marrow were pipetted and suspended in RPMI-1640 medium with 10% FBS to give single-cell suspension. Cells were then passed through a 100-μm cell strainer (Corning, New York, NY). For cell counting, the hemocytometer was used. For flow cytometry, fluorescence probes were added, and cells were incubated on ice for 1 h. For each flow cytometry experiment, unstained cell control and single-color compensation controls had been performed. After washing with PBS 3 times, bone marrow cells were examined on a FACSCalibur flow cytometer or LSRFortessa X-20 analyzer (BD Biosciences, San Jose, CA) and analyzed with FlowJo software (Tree Star, Inc., Ashland, OR). About 50,000 bone marrow cells were obtained for each analysis.

### Statistical analysis

Statistical significance was determined by one-way ANOVA (LSD Post Hoc Test) or Student's t test. P values < 0.05 were considered statistically significant.

## Results

### ^64^Cu-αCD11b radiotracer

The binding affinity of ^64^Cu labeled DOTA-αCD11b antibodies was determined by competitive displacement between ^64^Cu-αCD11b and cold αCD11b antibody or DOTA- αCD11b antibody in murine macrophage RAW264.7 cells. Both preparations of ^64^Cu-αCD11b with 2.54 or 5.77 DOTA moieties conjugated to each antibody maintained their binding affinity to CD11b (**Figure S1**).

### ^64^Cu-αCD11b µPET visualized myeloid cells in the bone marrow and spleen

We previously showed that ^64^Cu-αCD11b specifically binds to CD11b^+^ myeloid cells *in vitro* and *in vivo* in immunocompetent mice 7. In the current study, we first analyzed the biodistribution of ^64^Cu-αCD11b in non-tumor bearing immunodeficient nude mice. ^64^Cu-αCD11b was primarily localized to the bone marrow and spleen (**Figure S2A**). The bone marrow and spleen uptakes of ^64^Cu-αCD11b, derived by quantitative analysis of the μPET images, increased over time and plateaued at 4 h and 24 h after radiotracer injection, respectively. For quantitative analysis, the ROIs for the bone marrow compartment in the PET images were drawn on sacrum S1 spinal cord. The bone marrow uptakes of ^64^Cu-αCD11b were 18.9 ± 2.2, 26.1 ± 2.4, 22.7 ± 3.6, 16.3 ± 2.4, and 10.8 ± 1.4 %ID/g at 1, 4, 24, 48, and 72 h after injection, respectively (n = 3). The spleen uptakes of ^64^Cu-αCD11b were 19.5 ± 2.1, 22.1 ± 1.4, 24.1 ± 0.5, 21.2 ± 1.1, and 18.8 ± 3.22 at 1, 4, 24, 48, and 72 h after injection, respectively. The activity of ^64^Cu-αCD11b in all other major organs (e.g., heart, liver, and kidney) decreased steadily over time from 1 h to 72 h after radiotracer injection (**Figure S2B**). These results are in agreement with our previous findings in immunocompetent mice 7 and indicate that ^64^Cu-αCD11b is suitable for assessing response of bone marrow myeloid cells to chemotherapy in preclinical human tumor xenograft models.

To exclude the possibility of nonspecific uptake of antibody in bone marrow and spleen, we used ^64^Cu-IgG as a control. PET imaging showed low accumulation of ^64^Cu-IgG in the bone marrow and spleen (**Figure S3A**), with most of the signal being detected in the blood pool, heart and liver, a distribution pattern typical of nonspecific antibodies 14. Biodistribution results confirmed these findings and showed that the uptakes ^64^Cu-αCD11b were significantly higher than that of ^64^Cu-IgG in the bone marrow and spleen (p < 0.001) (**Figure S3B**).

### ^64^Cu-αCD11b µPET-CT revealed depletion of myeloid cells by single-dose Abraxane and prevention of myeloid cell depletion by αCSF-1 in the bone marrow and spleen

**Figure 1:**
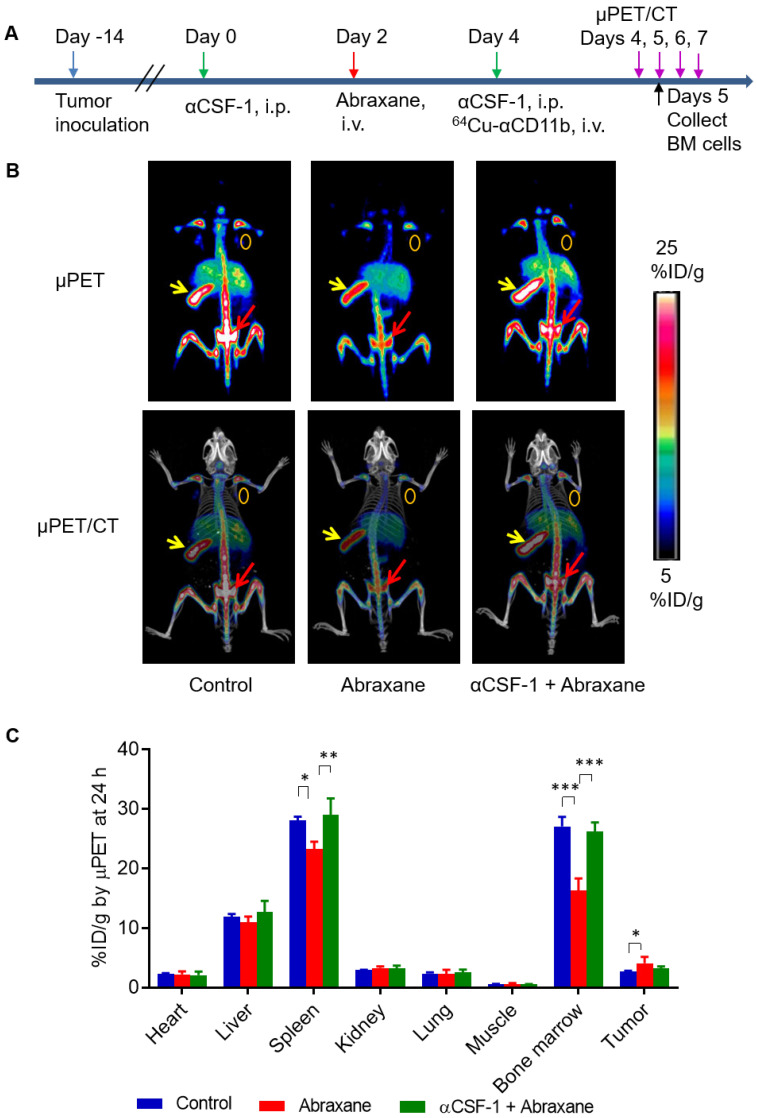
**^64^Cu-αCD11b µPET-CT of MDA-MB-435 tumor-bearing nude mice treated with a single dose of Abraxane.** Untreated mice were used as a control. (**A**) Scheme of experimental design. (**B**) Representative µPET-CT images acquired 24 h after intravenous injection of ^64^Cu-αCD11b. Red arrows: bone marrow; yellow arrows: spleen; gold circles: tumor. (**C**) Quantitative analysis of organ distribution of ^64^Cu-αCD11b from images acquired 24 h after radiotracer injection. Data are expressed as mean ± standard deviation (n = 3/group). *, p < 0.05; **, p < 0.01; ***, p < 0.001.

In the Abraxane single-dose regimen (**Figure 1A**), ^64^Cu-αCD11b µPET-CT showed significantly less uptake of ^64^Cu-αCD11b in the bone marrow and spleen of nude mice bearing MDA-MB-435 tumors after Abraxane injection than in the bone marrow and spleen of untreated mice. Combining αCSF-1 with Abraxane completely reversed the effect of Abraxane on myeloid cell depletion in bone marrow and spleen. Representative μPET and μPET-CT images acquired 24 h after ^64^Cu-αCD11b injection were shown in **Figure 1B** and **Figure S4**. The bone marrow uptakes of ^64^Cu-αCD11b in untreated, Abraxane-treated, and αCSF-1 plus Abraxane-treated mice were 27.0 ± 1.6, 16.3 ± 2.0, and 26.1 ± 1.6 %ID/g, respectively, 24 h after injection according to quantitative image analysis (**Figure 1C**). Abraxane significantly lowered the uptake of ^64^Cu-αCD11b in the bone marrow compared to the uptake in untreated mice (p < 0.001) and αCSF-1 plus Abraxane-treated mice (p < 0.001). The spleen uptake of ^64^Cu-αCD11b was also significantly lower in the Abraxane-treated mice (23.2 ± 1.3%ID/g) than in the untreated mice (28.1 ± 0.6%ID/g; p < 0.05) and the αCSF-1 plus Abraxane-treated mice (28.9 ± 2.8 %ID/g; p < 0.01) 24 h after injection (**Figure 1C**). In addition, there were no significant difference in the bone marrow and spleen uptakes of ^64^Cu-αCD11b between untreated and αCSF-1 alone treated mice (**Figure S5**).

### Flow cytometry confirmed depletion of myeloid cells by Abraxane and prevention of myeloid cell depletion by αCSF-1 in the bone marrow

**Figure 2:**
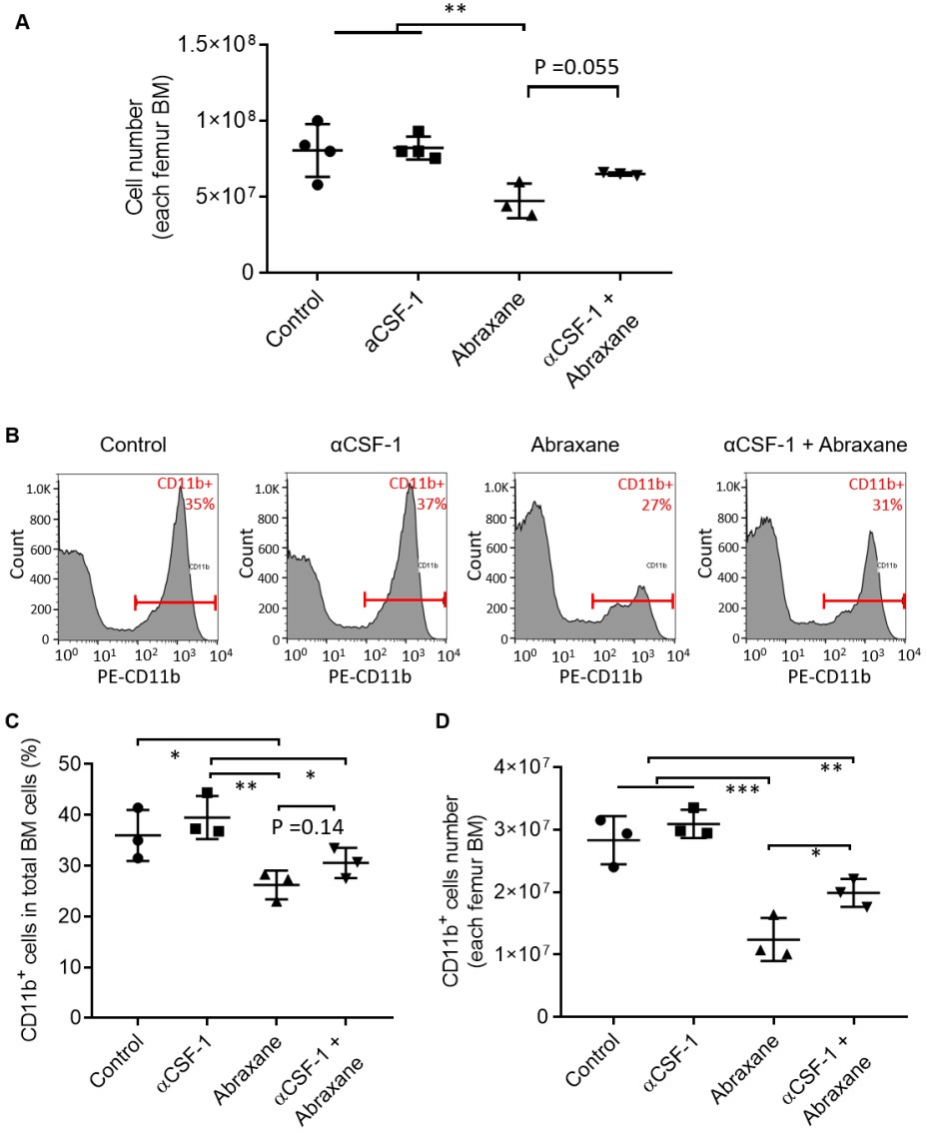
** Flow cytometry analysis of bone marrow cells from mice treated with αCSF-1, Abraxane, or αCSF-1 plus Abraxane.** Bone marrow cells were collected on day 5 as shown in Figure 1A. Untreated mice were used as a control. (**A**) Numbers of cells in femur bone marrow (BM) after different treatments. (**B-D**) Analysis of CD11b^+^ cell population in the bone marrow. (**B**) Representative flow cytometry histograms. (**C**) CD11b^+^ cells as a percentage of total bone marrow cells. (**D**) Number of CD11b^+^ cells in total aspirated bone marrow cells. Data are expressed as mean ± standard deviation (n = 3/group). *, p < 0.05; **, p < 0.01; ***, p < 0.001.

At 3 days after a single Abraxane injection (day 5, **Figure 1A**), the number of bone marrow nucleated cells in the femur was significantly lower in Abraxane-treated mice than in untreated mice (p < 0.01) (**Figure 2A**), indicating that Abraxane depleted bone marrow cells. Compared to Abraxane alone, αCSF-1 plus Abraxane increased bone marrow cellularity (p = 0.055). αCSF-1 alone did not affect bone marrow cellularity compared to no-treatment control, suggesting that CSF-1/CSF-1R blockade did not impact bone marrow proliferation during the time frame of this study (5 days) (**Figure 2A**).

To confirm these data, we further analyzed bone marrow CD11b^+^ cells by flow cytometry (**Figure 2B**). The CD11b^+^ populations in all bone marrow cells were 36.0 ± 5.0%, 39.5 ± 4.3%, 26.2 ± 2.8%, and 30.5 ± 3.0% for untreated, αCSF-1-treated, Abraxane-treated, and αCSF-1 plus Abraxane-treated mice, respectively. The CD11b^+^ cell population was higher in mice treated with αCSF-1 plus Abraxane than in mice treated with Abraxane alone (**Figure 2C**). The number of total CD11b^+^ cells in the femur bone marrow of mice in each treatment group is shown in **Figure 2D**. Abraxane strongly reduced the number of CD11b^+^ cells compared to the numbers of CD11b^+^ cells in the other 3 groups (**Figure 2D**). These data confirmed that ^64^Cu-αCD11b µPET-CT was indeed reporting changes in the CD11b^+^ population in the bone marrow after treatment with Abraxane and αCSF-1 plus Abraxane.

### αCSF-1 mitigated Abraxane-mediated depletion of bone marrow myeloid cells primarily through reducing depletion of Ly6G^+^Ly6C^low^ granulocytes

**Figure 3:**
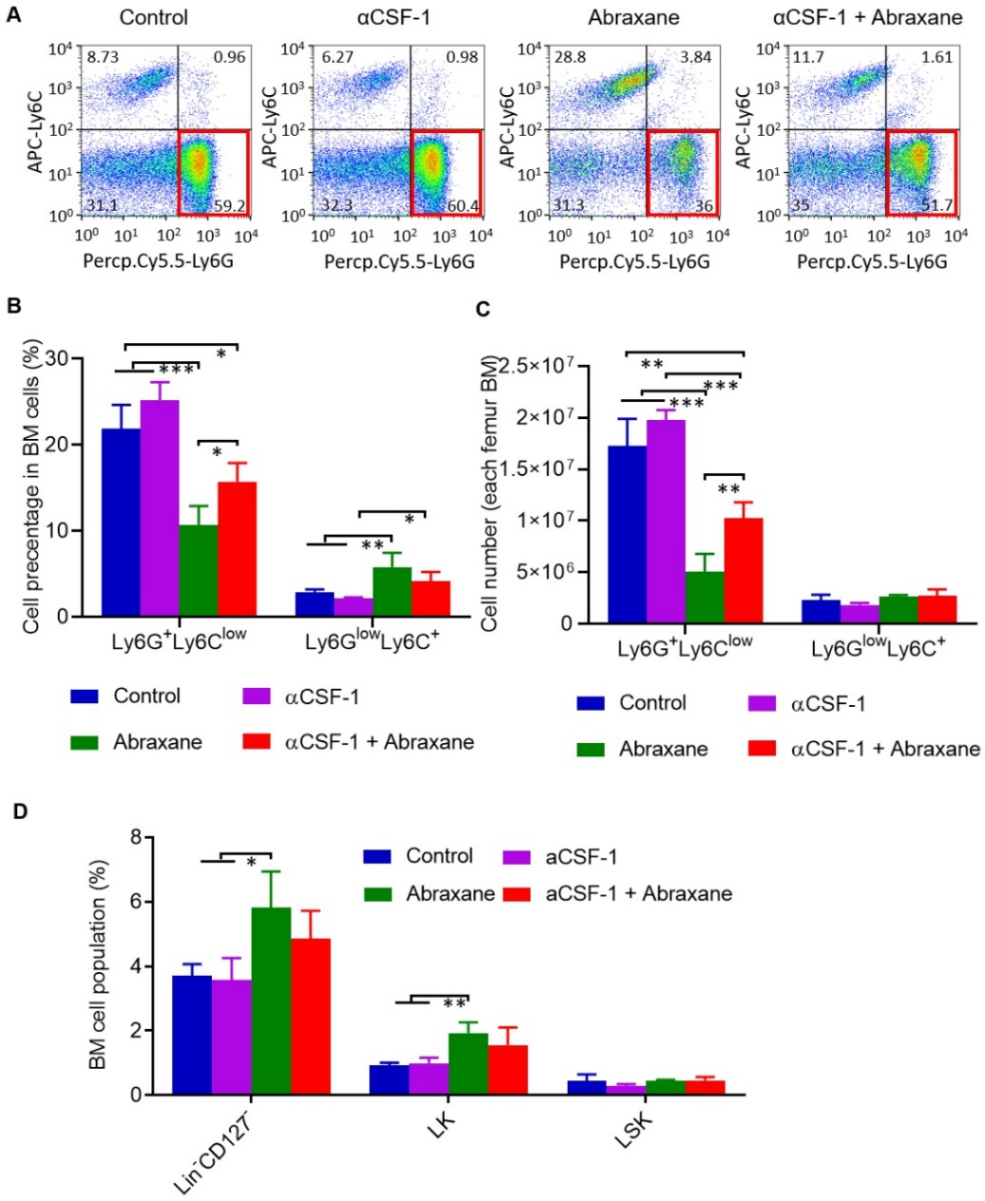
** Flow cytometry analysis of subpopulations of myeloid cells from bone marrow of mice treated with αCSF-1, Abraxane, or αCSF-1 plus Abraxane.** Bone marrow cells were collected on day 5 as shown in Figure 1A. Untreated mice were used as a control. (**A**) Representative dot plots of bone marrow cells stained with Ly6G and Ly6C in bone marrow CD11b^+^ gating cell population. (**B**) Quantification of Ly6G^+^Ly6C^low^ granulocytic myeloid cells and Ly6G^low^Ly6C^+^ monocytic myeloid cells as a percentage of all bone marrow (BM) cells. (**C**) Quantification of number of Ly6G^+^Ly6C^low^ granulocytic myeloid cells and Ly6G^low^Ly6C^+^ monocytic myeloid cells in all aspirated bone marrow cells. (**D**) Flow cytometry analysis of bone marrow populations of pluripotent hematopoietic stem cells (LSK cells) and myeloid progenitor cells (LK cells) after a single-dose Abraxane regimen shown in Figure 1A. Cells were collected on day 5. Data are expressed as mean ± standard deviation (n = 3/group). Lin, lineage. *, p < 0.05; **, p < 0.01, ***, p < 0.001.

There are 2 major subsets of CD11b^+^ myeloid cells in the bone marrow, Ly6G^+^Ly6C^low^ granulocytes and Ly6G^low^Ly6C^+^ monocytes 15. Using cell gating strategy shown in **Figure S6**, analysis of subpopulations of CD11b^+^ gating myeloid cells taken on day 5 (**Figure 1A**) revealed that Ly6G^+^Ly6C^low^ granulocytes but not Ly6G^low^Ly6C^+^ monocytes were strongly suppressed by Abraxane and that αCSF-1 partially mitigated Abraxane-mediated depletion of Ly6G^+^Ly6C^low^ cells (**Figure 3A**). The populations of Ly6G^low^Ly6C^+^ cells as a percentage of all bone marrow cells were 2.9 ± 0.3%, 2.1 ± 0.2%, 5.8 ± 1.7%, and 4.2 ± 1.1% for untreated, αCSF-1-treated, Abraxane-treated, and αCSF-1 plus Abraxane-treated mice, respectively. The populations of Ly6G^+^Ly6C^low^ cells as a percentage of all bone marrow cells were 21.9 ± 2.79%, 25.2 ± 2.08%, 10.7 ± 2.16%, and 15.7 ± 2.21% for untreated, αCSF-1-treated, Abraxane-treated, and αCSF-1 plus Abraxane-treated mice, respectively (**Figure 3B**). The total numbers of Ly6G^+^Ly6C^low^ cells and Ly6G^low^Ly6C^+^ cells from femur bone marrow of 4 treatment groups are presented in **Figure 3C**. There were significantly fewer Ly6G^+^Ly6C^low^ granulocytes in the bone marrow of Abraxane-treated mice compared to untreated mice (p < 0.001) and αCSF-1 plus Abraxane-treated mice (p < 0.05). These data show that αCSF-1 could mitigate the loss of bone marrow granulocytes caused by Abraxane.

To confirm these findings, bone marrow cells from nude mice were directly incubated with αCSF-1 in the presence and absence of Abraxane. Flow cytometry analysis showed that the populations of Ly6G^low^Ly6C^+^ and Ly6G^+^Ly6C^low^ cells as a percentage of all bone marrow cells changed with the same trend as that of bone marrow cells collected and analyzed after treatments in mice (**Figure S7**).

Bone marrow myeloid cells originate from pluripotent hematopoietic stem cells (lineage^neg^/CD127^-^/CD117^+^/Sca1^+^; LSK cells). LSK cells can be further differentiated into lineage^neg^/CD127^-^/CD117^+^/Sca1^-^ myeloid progenitor populations (LK cells) and lineage^neg^/CD127^+^/CD117^+^/Sca1^+^ common lymphoid progenitors. To assess whether Abraxane depleted LSK and LK cells and whether αCSF-1 mitigated such an effect, we analyzed changes in the populations of lineage^neg^/CD127^-^ (LSK plus LK) cells. Abraxane-treated mice had significantly more lineage^neg^/CD127^-^ cells in the bone marrow than untreated mice and αCSF-1-treated mice (p < 0.05). αCSF-1 plus Abraxane also caused a moderate increase in the lineage^neg^/CD127^-^ population compared to no-treatment control (p = 0.126) and αCSF-1 (p = 0.072). Moreover, the populations of LK cells in the bone marrow were significantly higher in Abraxane-treated mice than in untreated and αCSF-1-treated mice (p < 0.01) (**Figure 3D**). These data suggest that the myeloid progenitor cells in the bone marrow of Abraxane-treated mice were activated to replenish the depleted CD11b^+^ myeloid cells and Ly6G^+^Ly6C^low^ granulocytes, and that αCSF1 alone did not impact on bone marrow stem cell and progenitor cell biology.

### αCSF-1 inhibited Abraxane-mediated recruitment of macrophages to the tumor

**Figure 4:**
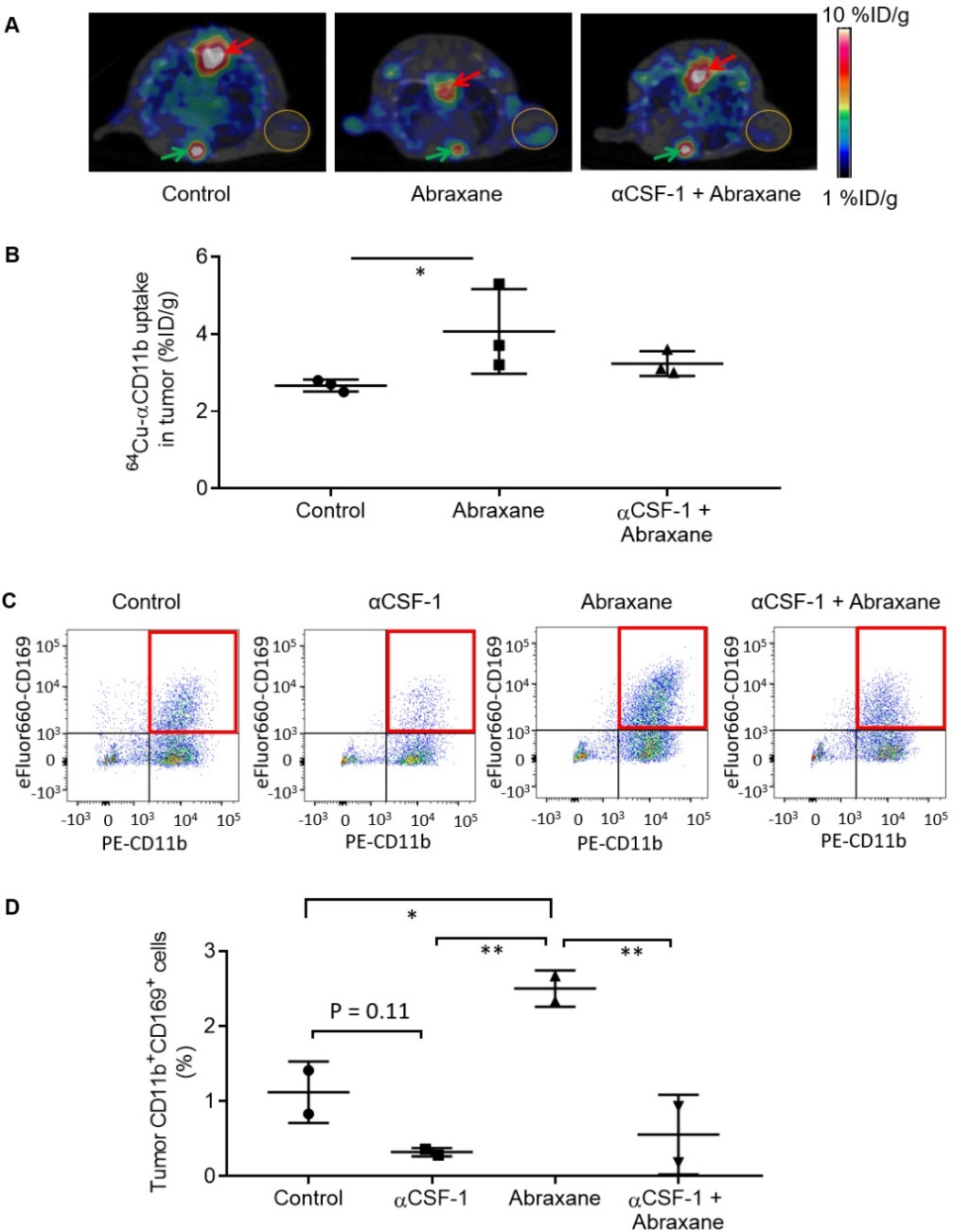
** Inhibition of Abraxane-mediated recruitment of macrophages to the tumor by αCSF-1.** (**A**) ^64^Cu-αCD11b µPET-CT of MDA-MB-435 tumor-bearing female nude mice treated with the single-dose Abraxane regimen as shown in Figure 1A. Images were acquired 24 h after radiotracer injection. Red arrows: bone marrow of thoracic spine; green arrows: bone marrow of sternum; gold circles: tumor. (**B**) Quantitative analysis of tumor uptake of ^64^Cu-αCD11b from images acquired 24 h after radiotracer injection. (**C**) Representative dot plots of CD45^+^ cells in the tumors stained with CD11b and CD169. (**D**) Quantification of CD11b^+^CD169^+^ macrophages in CD45^+^ cells in the tumors. Data are expressed as mean ± standard deviation (n = 3/group). *, p < 0.05; **, p < 0.01.

The uptake of ^64^Cu-αCD11b in MDA-MB-435 tumors could be visualized by μPET-CT at a lowered scale range (1 - 10 %ID/g) (**Figure 4A**). Multi-modality images showed that the tumor-infiltrating macrophages were primarily located at the junction between the viable tumor zone and the apoptotic cell death zone 16. Similarly, the intratumoral distribution of ^64^Cu-αCD11b in MDA-MB-435 tumors was heterogeneous. Quantification of imaging data showed that the tumor uptakes of ^64^Cu-αCD11b in the untreated, Abraxane-treated, and αCSF-1 plus Abraxane-treated mice were 2.67 ± 0.15, 4.07 ± 1.10, and 3.23 ± 0.32 %ID/g, respectively, 24 h after injection (**Figure 4B**). Abraxane increased uptake of ^64^Cu-αCD11b in the tumor compared to no treatment (p < 0.05). Tumor uptake of ^64^Cu-αCD11b was moderately lower in the αCSF-1 plus Abraxane-treated tumors than in the Abraxane-treated tumors (p = 0.176). These results are consistent with previously reported data, which showed that chemotherapy could increase tumor infiltration of macrophages 16-18 while αCSF-1 could deplete macrophages residing in the tumor 19.

Consistent with the μPET imaging data, the population of CD11b^+^CD169^+^ macrophages in CD45^+^ cells was significantly higher in the Abraxane-treated tumors than that in the untreated tumors (p < 0.05), the αCSF-1-treated tumors (p < 0.01), and the αCSF-1 plus Abraxane-treated tumors (p < 0.01) (**Figure 4C - D**). These data demonstrated that αCSF-1 could prevent macrophage recruitment to the tumor microenvironment after Abraxane chemotherapy.

### ^64^Cu-αCD11b µPET-CT of myeloid cells after multiple doses of Abraxane and αCSF-1

**Figure 5:**
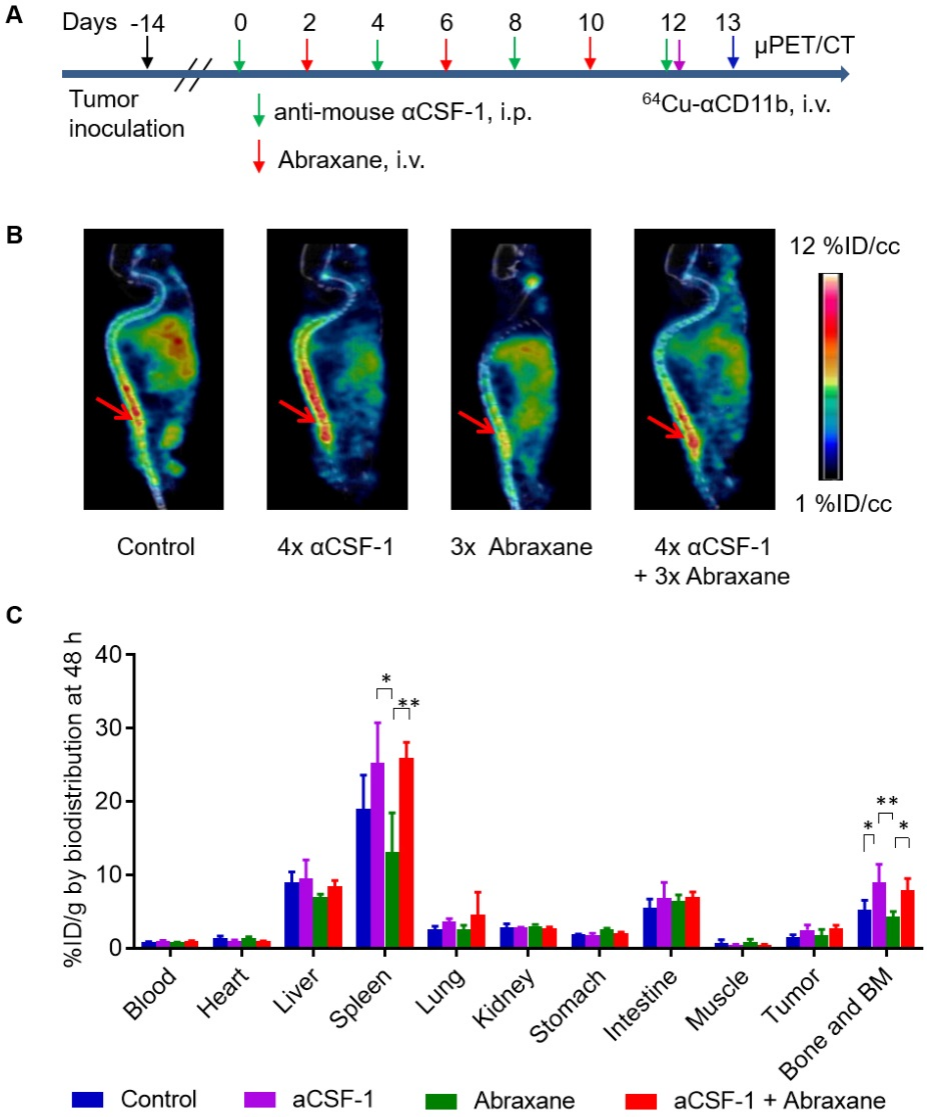
**^64^Cu-αCD11b µPET-CT of MDA-MB-435 tumor-bearing female nude mice treated with a multidose Abraxane regimen.** Untreated mice were used as a control. (**A**) Scheme of experimental design. (**B**) Representative µPET-CT images acquired 24 h after intravenous injection of ^64^Cu-αCD11b. Red arrows: bone marrow. (**C**) Biodistribution of ^64^Cu-αCD11b 48 h after ^64^Cu-αCD11b injection. Data are expressed as mean ± standard deviation (n = 3/group). *, p < 0.05; **, p < 0.01.

In the Abraxane multidose treatment regimen (**Figure 5A**), ^64^Cu-αCD11b μPET revealed that compared to no treatment control, 4 doses of αCSF-1 over a span of 13 days significantly increased bone marrow uptake of ^64^Cu-αCD11b 24 h after injection, suggesting either increased hematopoiesis or decreased mobilization of myeloid cells out of bone marrow. Given that αCSF-1 did not impact on proliferation of bone marrow stem cells and progenitor cells, it is likely that αCSF-1 suppressed myeloid cell trafficking, resulting in accumulation of these cells in the bone marrow after a multidose regimen. In comparison, 3 doses of Abraxane in the same time period moderately reduced bone marrow uptake of ^64^Cu-αCD11b. Combined αCSF-1 plus Abraxane protected Abraxane induced myeloid cell depletion in bone marrow (**Figure 5B**).

These observations were confirmed by the classic cut-and-count method (n = 3/group). Consistent with the µPET data, the uptakes of ^64^Cu-αCD11b in the bone marrow of untreated, αCSF-1-treated, Abraxane-treated, and αCSF-1 plus Abraxane-treated mice were 5.23 ± 1.33, 9.02 ± 2.42, 4.33 ± 0.70, and 7.93 ± 1.58 %ID/g, respectively, 48 h after injection (**Figure 5C**). The bone marrow uptakes of ^64^Cu-αCD11b in the αCSF-1-treated (p < 0.01) and αCSF-1 plus Abraxane-treated mice (p < 0.05) were significantly higher than that in the Abraxane-treated mice. Unlike what was observed with the Abraxane single-dose regimen, the bone marrow uptake of ^64^Cu-αCD11b in the Abraxane-multi-dose treated mice was only moderately lower than that in the untreated mice (p = 0.36), suggesting activation of a compensatory mechanism to replenish bone marrow cells after the initiation of Abraxane treatments in the multidose regimen.

The spleen uptakes of ^64^Cu-αCD11b in the control, αCSF-1, Abraxane, and αCSF-1 plus Abraxane treatment groups were 19.0 ± 4.7, 25.3 ± 5.5, 13.1 ± 5.4, and 26.0 ± 2.1 %ID/g, respectively, 48 h after injection (**Figure 5C**). The spleen uptakes of ^64^Cu-αCD11b in the αCSF-1-treated mice (p < 0.05) and αCSF-1 plus Abraxane-treated mice (p < 0.01) were significantly higher than that in the Abraxane-treated mice.

### ^64^Cu-αCD11b µPET-CT revealed depletion of myeloid cells in the bone marrow by Abraxane and preservation of myeloid cells in the bone marrow by anti-human αCSF-1 in transgenic mice expressing human CSF-1

**Figure 6:**
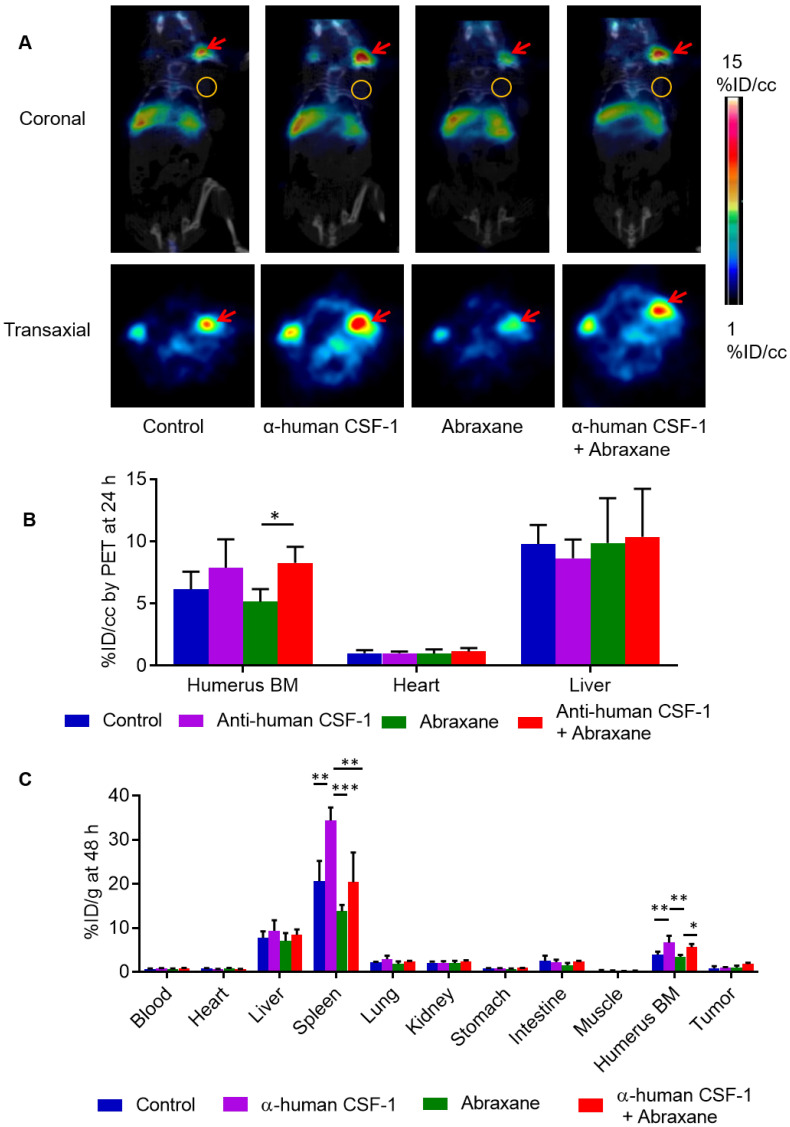
** µPET-CT and biodistribution of ^64^Cu-αCD11b in transgenic JAX CSF-1 knock-in mice expressing human CSF-1.** Mice were treated with anti-human (α-human) CSF-1, Abraxane, or anti-human CSF-1 plus Abraxane. Untreated mice were used as a control. (**A**) Representative ^64^Cu-αCD11b µPET-CT images of JAX CSF-1 knock-in mice bearing subcutaneous MDA-MB-435 tumors acquired 24 h after intravenous injection of radiotracer. Red arrows: humerus bone marrow; gold circles: tumor. (**B**) Quantitative analysis of tissue uptake of ^64^Cu-αCD11b from μPET images obtained 24 h after radiotracer injection. (**C**) Biodistribution of ^64^Cu-αCD11b obtained 48 h after radiotracer injection. Data are expressed as mean ± standard deviation (n = 3/group). *, p < 0.05; **, p < 0.01; ***, p < 0.001.

Several CSF-1/CSF-1R signaling inhibitors have entered into clinical trials 20. To characterize the effect of anti-human CSF-1 antibody on bone marrow uptake of ^64^Cu-αCD11b, we used transgenic JAX CSF-1 knock-in mice that express functional human CSF-1 11. In contrast with the pattern of distribution of ^64^Cu-αCD11b in nude mice (**Figure S2A**), ^64^Cu-αCD11b was primarily accumulated in humerus bone marrow in JAX CSF-1 knock-in mice, with a relatively low uptake in other parts of the skeletal system (**Figure 6A**). ^64^Cu-αCD11b µPET-CT showed that the bone marrow uptake of ^64^Cu-αCD11b was lower in mice treated with 3 doses of Abraxane than in untreated control mice and higher in mice treated with 4 doses of anti-human CSF-1 antibody than in untreated control mice. Moreover, combined anti-human CSF-1 antibody plus Abraxane reversed depletion of bone marrow CD11b^+^ myeloid cells caused by Abraxane (**Figure 6A**). The bone marrow uptakes of ^64^Cu-αCD11b in untreated mice and mice treated with anti-human CSF-1 antibody, Abraxane, and anti-human CSF-1 plus Abraxane were 6.15 ± 1.43, 7.90 ± 2.28, 5.16 ± 1.02, and 8.28 ± 1.30 %ID/cc, respectively, based on quantitative analysis of μPET-CT images acquired 24 h after injection, which was 13 days after initiation of treatments. Combining anti-human CSF-1 with Abraxane significantly increased bone marrow uptake of ^64^Cu-αCD11b compared to treatment with Abraxane alone (p < 0.05) (**Figure 6B**).

Biodistribution data obtained by the cut-and-count method at the end of the imaging session showed that consistent with the µPET-CT data, the uptakes of ^64^Cu-αCD11b in the bone marrow of untreated mice and mice treated with anti-human CSF-1, Abraxane, and anti-human CSF-1 plus Abraxane were 3.98 ± 0.66, 6.65 ± 1.55, 3.47 ± 0.41, and 5.61 ± 0.73 %ID/g, respectively (**Figure 6C**). The bone marrow uptake of ^64^Cu-αCD11b was significantly higher in the mice treated with anti-human CSF-1 plus Abraxane than in the mice treated with Abraxane alone (p < 0.05) and significantly higher in the mice treated with anti-human CSF-1 than in the untreated mice and Abraxane-treated mice (p < 0.01). The uptakes of ^64^Cu-αCD11b in the spleen of untreated mice and mice treated with anti-human CSF-1, Abraxane, and anti-human CSF-1 plus Abraxane were 20.6 ± 4.6, 34.3 ± 2.97, 13.8 ± 1.4, and 20.4 ± 6.8 %ID/g, respectively (**Figure 6C**). The uptake of ^64^Cu-αCD11b in the spleen was significantly higher in anti-human CSF-1-treated mice than in untreated mice (p < 0.01), Abraxane-treated mice (p < 0.001), and anti-human CSF-1 plus Abraxane-treated mice (p < 0.01). The addition of anti-human CSF-1 prevented depletion of spleen myeloid cells by Abraxane (p = 0.11).

## Discussion

In the present study, we found that ^64^Cu-αCD11b μPET-CT could accurately delineate depletion of bone marrow myeloid cells by Abraxane chemotherapy and that αCSF-1 could mitigate Abraxane-induced depletion of bone marrow cells. These findings suggest that quantitative ^64^Cu-αCD11b μPET-CT is a useful tool for assessing engagement of CSF-1/CSF-1R signal-blocking agents used in combination with chemotherapy. Our data also suggest that longitudinal imaging of CD11b^+^ cells might have a role in assessing trafficking of bone marrow derived myeloid cells under various disease conditions including tumors and inflammatory diseases, and in monitoring response of these diseases to various treatments.

^64^Cu-αCD11b μPET-CT acquired on day 5 showed that a single dose of Abraxane caused significant reduction of ^64^Cu-αCD11b uptake in the bone marrow and spleen of nude mice. Abraxane, as a cremophor-free and albumin-bound nanoparticle, can enhance the therapeutic effectiveness by increasing intratumor paclitaxel concentrations and endothelial cell transport 21. We previously reported that Abraxane treatment induced apoptosis of MDA-MB-435 tumors 16. Cells undergoing apoptosis express specific “eat me” signals (e.g. phosphatidylserine) that facilitate recognition and ingestion by macrophages 22. As a result, Abraxane treatment results in tumor infiltration of macrophages. A common side effect of paclitaxel is bone marrow depression. In this study, the depletion of bone marrow cells by Abraxane was confirmed by counting total cell number in femur and by flow cytometry analysis of CD11b^+^ myeloid cells. ^64^Cu-αCD11b μPET-CT acquired on day 13 after the first of 3 injections of Abraxane also showed a small reduction in bone marrow uptake of the radiotracer, although the difference was not statistically significant. The main CD11b^+^ cell population in the bone marrow depleted by Abraxane was Ly6G^+^Ly6C^low^ granulocytes, with minimal effect on Ly6G^low^Ly6C^+^ monocytes. It is possible that other cell types in the bone marrow (i.e. CD11b^+^Ly6G^low/-^Ly6C^low/-^ cells) may also be affected by Abraxane treatment. Interestingly, the populations of lineage^neg^/CD127^-^ cells and lineage^neg^/CD127^-^/CD117^+^/Sca1^-^ myeloid progenitor LK cells were increased 3 days after a single Abraxane injection compared to the populations in untreated mice and αCSF-1-treated mice, suggesting that the initial insult by Abraxane activated a compensatory mechanism to replenish bone marrow cells. αCSF-1 alone did not affect populations of LK and LKS cells.

Compared to Abraxane alone, combined αCSF-1 plus Abraxane better maintained CD11b^+^ cell population in the bone marrow and spleen. Our imaging studies in both single-dose and multidose settings showed that combining αCSF-1 with Abraxane significantly reduced Abraxane-induced loss of myeloid cells in the bone marrow and spleen. Further analysis showed that compared to Abraxane alone, αCSF-1 plus Abraxane increased bone marrow cellularity, increased the CD11b^+^ cell population in the bone marrow, and increased the Ly6G^+^Ly6C^low^ granulocyte population in the bone marrow. Abraxane and αCSF-1 plus Abraxane increased bone marrow lineage^neg^/CD127^-^ cells and myeloid progenitor LK cells compared to the levels in untreated mice and αCSF-1-treated mice, suggesting that the increase in bone marrow myeloid cells by αCSF-1 was not a result of activation of the self-renewal of the LSK cells or increased proliferation of the LK cells. Rather, it is likely that CSF-1/CSF-1R blockade by αCSF-1 inhibited signaling for myeloid cells to exit the bone marrow compartment, resulting in accumulation of these cells in the bone marrow in the multidose setting. A mechanism like what was observed in the bone marrow may explain depletion of CD11b^+^ myeloid cells in the spleen after Abraxane chemotherapy, and recovery of CD11b^+^ myeloid cells in the spleen after αCSF-1 immunotherapy. Taken together, we conclude that ^64^Cu-αCD11b μPET-CT is useful for simultaneous evaluation of the effects of chemotherapy and immunotherapy at both the local tumor level and the whole-body level in the bone marrow and spleen.

The goal of CSF-1/CSF-1R blockade is to reduce accumulation of immunosuppressive tumor-associated macrophages and MDSCs in the tumor microenvironment 19. Tumor-infiltrating myeloid cells have been recognized as important mediators of not only tumor progression and metastasis 23, 24 but also therapeutic resistance 25-28. Both chemotherapy and radiation therapy have been shown to result in increased tumor infiltration of macrophages and MDSCs 16-18, 29, 30. In the bone marrow, CSF-1/CSF-1R signaling promotes the differentiation of myeloid progenitors into heterogeneous populations of monocytes, granulocytes, macrophages, dendritic cells, and bone-resorbing osteoclasts. In the blood circulation and tumor microenvironment, CSF-1 regulates the migration and function of these cells 31. In mammary carcinoma-bearing transgenic PyMT mice, CSF-1 was upregulated after exposure to paclitaxel, which was believed to contribute to the recruitment of macrophages to the tumor; combined αCSF-1 and paclitaxel significantly delayed tumor growth 4. Consistent with this finding, we observed that Abraxane increased tumor infiltration of CD45^+^ hematopoietic cells and CD11b^+^CD169^+^ macrophages in the MDA-MB435 tumors compared to untreated tumors and αCSF-1-treated tumors. In contrast, αCSF-1 reduced infiltration of CD45^+^ hematopoietic cells and CD11b^+^CD169^+^ macrophages in tumors. Combined αCSF-1 plus Abraxane significantly decreased the population of CD11b^+^CD169^+^ cells in the tumors compared to Abraxane-treated tumors (**Figure 4C-D**). The opposite directions of change in CD11b^+^ cells in the bone marrow and tumors after Abraxane treatment (decrease in bone marrow and increase in tumors) and αCSF-1 (increase in bone marrow and decrease in tumors) indicate that CSF-1/CSF-1R blockade by αCSF-1 impacted trafficking of CD11b^+^ cells from the bone marrow to tumors.

It is worth noting that changes in bone marrow myeloid cells following treatment with anti-human CSF-1 and combined anti-human CSF-1 antibody plus Abraxane were readily visualized in a transgenic mouse model that expresses human CSF-1, suggesting that ^64^Cu-αCD11b PET-CT may be useful in evaluation of CSF-1/CSF-1R signaling blockade in the clinic. Several CSF-1/CSF-1R signaling inhibitors are currently being tested in clinical trials 20. At present, it is difficult to identify patients who might benefit from targeted CSF-1/CSF-1R blockade therapy. Noninvasive imaging of myeloid cells that illuminates the effects of CSF-1/CSF-1R blockade on the bone marrow, spleen, and tumor may improve understanding of the engagement of CSF-1/CSF-1R pathway inhibitors in patients, thereby facilitating the development of optimal combination strategies.

With the rapid development of immunoPET, CD11b has emerged as an important target for noninvasive characterization of myeloid cells. Rashidian et al. 32 used a much smaller variable domain of a camelid heavy-chain-only antibody targeting CD11b (~15 kDa), ^18^F-VHHDC13, which showed uptake in spleen and lymph nodes of mice. ^18^F-VHHDC13 could detect inflammation and infiltration of immune cells in tumor. Interestingly, ^18^F-VHHDC13 was very low uptake by bone marrow cells. Nigam et al. 33 recently demonstrated the potential for non-invasive quantification of tumor-infiltrating CD11b^+^ immune cells in a mouse model of glioma using PET with ^89^Zr-deferoxamine chelated anti-CD11b of the same clone (clone M1/70). ^89^Zr-deferoxamine-anti-CD11b showed significant uptake in the liver (~50%ID/g) and the spleen (>50%ID/g) at 72 h after injection. In comparison, the uptakes of ^64^Cu-anti-CD11b in the liver and the spleen at 72 h post-injection were 8.5 %ID/g and 18.8 %ID/g, respectively. These data suggest that 1) the Fc region of anti-CD11b may be important for its bone marrow homing activity, and 2) radiolabeling technique (the choice of radionuclide and radiometal chelator) could have significant impact on the biodistribution pattern of the anti-CD11b antibody.

Our study has a few limitations. First, CD11b is expressed on several different types of myeloid cells, and on a few non-myeloid cells as well. Additional imaging markers are needed in order to enhance cellular resolution. Second, lymphocytes and other cells usually account for ~20% of the bone marrow cells, there will be some limitations with ^64^Cu-αCD11b monitoring bone marrow disorders of originating from lymphocytes.

## Conclusions

^64^Cu-αCD11b is a useful PET imaging agent for monitoring chemotherapy-induced bone marrow suppression and mitigation of chemotherapy-induced bone marrow suppression by αCSF-1. Remarkably, αCSF-1 as part of combination therapy not only can enhance the efficacy of chemotherapy by blocking tumor infiltration of myeloid cells but also has the potential to preserve the bone marrow myeloid population and thus alleviate chemotherapy-induced bone marrow suppression.

## Supplementary Material

Supplementary figures and tables.

## Abbreviations

^64^Cu-αCD11b: ^64^Cu-DOTA-anti-CD11b
αCSF-1: anti-CSF-1 antibody
MDSCs: myeloid-derived suppressor cells
M-CSF: Macrophage colony-stimulating factor
PET-CT: positron emission tomography-computed tomography
BM: bone marrow
