# Supplementary material for: Abraxane-induced bone marrow CD11b+ myeloid cell depletion in tumor-bearing mice is visualized by μPET-CT with 64Cu-labeled anti-CD11b and prevented by anti-CSF-1
Source: Theranostics. 2021 Jan 20;11(7):3527–39. doi: 10.7150/thno.49421 (PMC7847669; doi:10.7150/thno.49421)
Supplement: Supplementary file 1 — Supplementary figures and tables. [file thnov11p3527s1.pdf]

Supplementary Data

Abraxane-induced bone marrow CD11b$^+$ myeloid cell depletion in tumor-bearing mice is visualized by µPET-CT with ^{64}Cu-labeled anti-CD11b and prevented by anti-CSF-1

Qizhen Cao[1], Qian Huang[1], Y. Alan Wang[2], and Chun Li[1]

Departments of [1]Cancer Systems Imaging and [2]Cancer Biology, The University of Texas MD Anderson Cancer Center, Houston, TX 77054

Corresponding author: Chun Li, PhD, Department of Cancer Systems Imaging, 1881 East Road-Unit 1907, The University of Texas MD Anderson Cancer Center, Houston, Texas 77054, USA; Tel: 713-792-5182; Fax: 713-794-5456; E-mail: cli@mdanderson.org.

Table of Contents

Materials and Methods

Table S1. Number of DOTA per αCD11b antibody on DOTA-αCD11b conjugate.

Figure S1. Cell binding assay.

Figure S2. μPET of female nude mice with ^{64}Cu-αCD11b at different time points.

Figure S3. μPET and biodistribution of ^{64}Cu-αCD11b or ^{64}Cu-IgG in normal female 129×1/svJ mice.

Figure S4. ^{64}Cu-αCD11b μPET-CT of MDA-MB-435 tumor-bearing nude mice treated with a single dose of Abraxane in low scale bar.

Figure S5. ^{64}Cu-αCD11b μPET and biodistribution of female nude mice without or with αCSF-1 treatment.

Figure S6. Gating strategy for flow cytometry analysis of bone marrow cell populations.

Figure S7. Flow cytometry analysis of bone marrow cells treated with αCSF-1 and/or Abraxane *in vitro*.

MATERIALS AND METHODS

DOTA conjugation and radiolabeling of IgG

p-SCN-Bn-DOTA was added to rat IgG2b (BioXCell, West Lebanon, NH) at a molar ratio of 50 : 1 in 0.1 M sodium bicarbonate buffer (pH 8.5). The resulting conjugate, DOTA-IgG, was purified by PD-10 column and concentrated by Centricon filter (Millipore, Bedford, MA). For radiolabeling, $^{64}CuCl_2$ was diluted with 0.2 mL of 0.1 M sodium acetate buffer, and the pH of the solution was adjusted to pH 6.0 with 1 N NaOH. DOTA-IgG (10 µg) was then added into sodium acetate-buffer solution containing 37 MBq of $^{64}CuCl_2$ and incubated for 1 h at 38 °C with constant shaking. The resulting ^{64}Cu-DOTA-IgG (^{64}Cu-IgG) was purified by PD-10 column using phosphate-buffered saline (PBS) as the mobile phase.

Number of DOTA per αCD11b Antibody on DOTA-αCD11b conjugate

The average number of DOTA chelators per αCD11b antibody was measured following reported procedures [1, 2]. Briefly, nonradioactive $CuCl_2$ (80-fold excess of DOTA-αCD11b) in 20 µL 0.1N sodium acetate (NaOAc) buffer (pH 5.5) was added to approximately 1.0 mCi $^{64}CuCl_2$ in 50 µL 0.1N NaOAc buffer, then, 20 µg of DOTA-αCD11b in 40 µL 0.1N NaOAc buffer were added to the above carrier-added $^{64}CuCl_2$ solution. The reaction mixture was incubated with constant shaking at 40 °C for 1 h. The resulting ^{64}Cu-DOTA-αCD11b (^{64}Cu-αCD11b) was purified by PD-10 column with 1 × PBS, and eluent (3.0–4.5 mL) was collected and counted for radioactivity. The number of DOTA per αCD11b antibody was calculated using the following equation: number of DOTA per αCD11b antibody = moles (Cu^{2+}) × activity (3.0–4.5 mL) / moles (DOTA-αCD11b) / total activity (loaded for each labeling). The activities in the equation were all decay-corrected to the same time point. The results were expressed as mean ± SD (n = 3).

Competitive cell-binding assay

RAW264.7 cells (murine macrophage cell line) were suspended in PBS containing 1% bovine serum albumin (1×10^5 cells per 50 µL). Cells were incubated with ^{64}Cu-αCD11b (0.1 µCi/well, ~$4\text{x}10^{-10}$ M) (2.54 ± 0.28 ^{64}Cu-DOTA moieties per αCD11b or 5.77 ± 0.39 ^{64}Cu-DOTA moieties per αCD11b) in the absence and presence of increasing concentrations of nonradioactive αCD11b or DOTA-αCD11b (2.54 DOTA per αCD11b or 5.77 DOTA per αCD11b) at room temperature for 2 h with gentle shaking. After removal of culture medium under vacuum, cells were washed 3 times with PBS containing 0.1% bovine serum albumin. Radioactivity of the cells from each well was counted with a gamma counter. The 50% inhibitory concentration of nonradioactive αCD11b was calculated by fitting the data with nonlinear regression using GraphPad Prism (GraphPad Software, La Jolla, CA).

Supplemental Table 1. Number of DOTA per αCD11b antibody on DOTA-αCD11b conjugate

DOTA/ αCD11b ratio	20 : 1	50 : 1
Number of DOTA per αCD11b	2.54 ± 0.28	5.77 ± 0.39

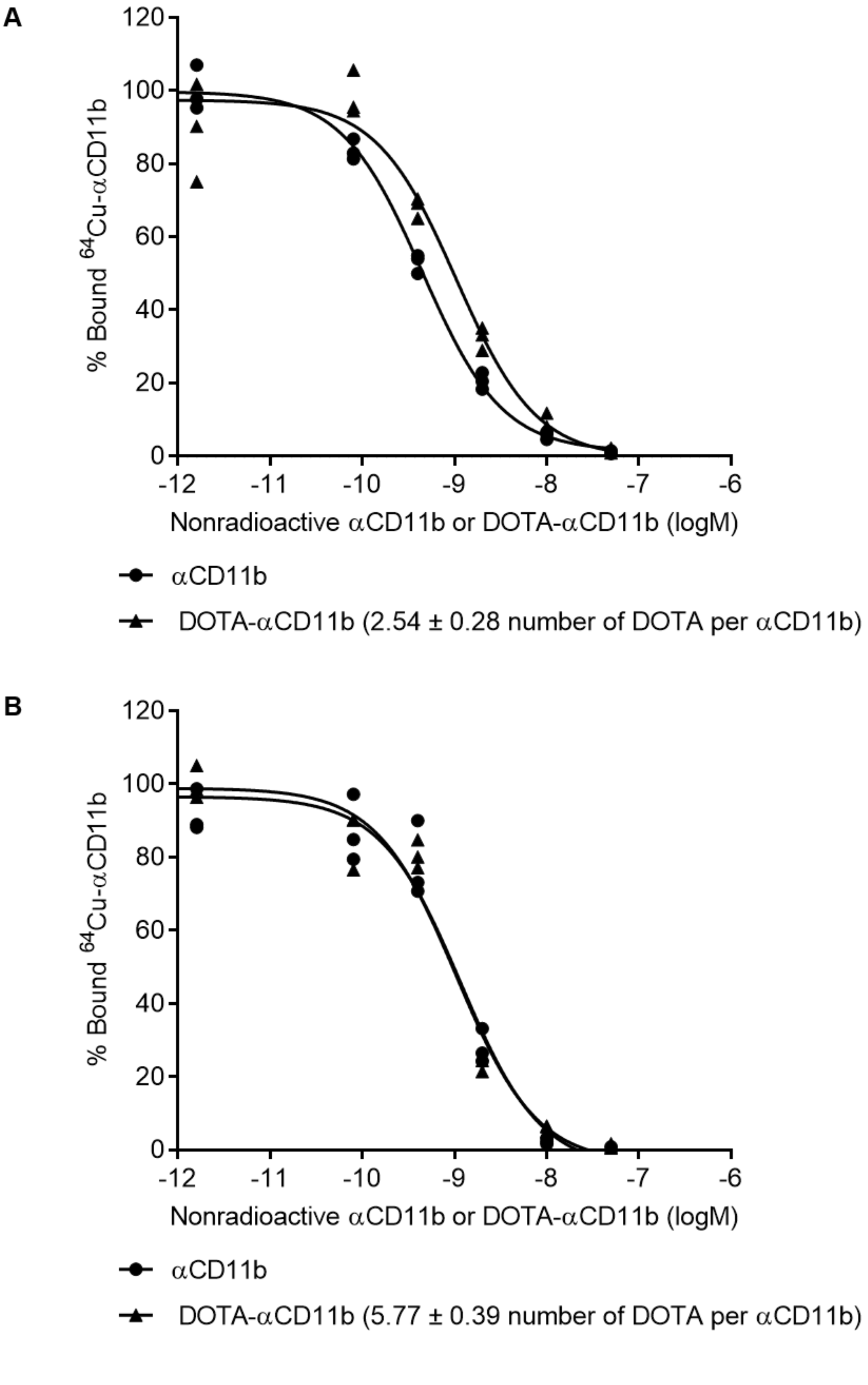

Figure S1. Cell binding assay. Displacement of the binding of ^{64}Cu-αCD11b to RAW264.7 cells by

nonradioactive αCD11b or DOTA-αCD11b (n = 3). (**A**) Displacement of the binding of ^{64}Cu-αCD11b (^{64}Cu labelled DOTA-αCD11b with 2.54 ± 0.28 number of DOTA per αCD11b) to RAW264.7 cells by nonradioactive αCD11b or DOTA- αCD11b (2.54 ± 0.28 number of DOTA per αCD11b). The 50% inhibitory concentration (IC50) between ^{64}Cu-αCD11b and nonradioactive αCD11b was 4.46×10^{-10} mol/L, the IC50 between ^{64}Cu-αCD11b and nonradioactive DOTA-αCD11b was 1.04×10^{-9} mol/L.

(**B**) Displacement of the binding of ^{64}Cu-αCD11b (^{64}Cu labelled DOTA-αCD11b with 5.77 ± 0.39 number of DOTA per αCD11b) to RAW264.7 cells by nonradioactive αCD11b or DOTA- αCD11b (5.77 ± 0.39 number of DOTA per αCD11b). The 50% inhibitory concentration (IC50) between ^{64}Cu-αCD11b and nonradioactive αCD11b was 1.17×10^{-9} mol/L, the IC50 between ^{64}Cu-αCD11b and nonradioactive DOTA-αCD11b was 1.05×10^{-9} mol/L.

A

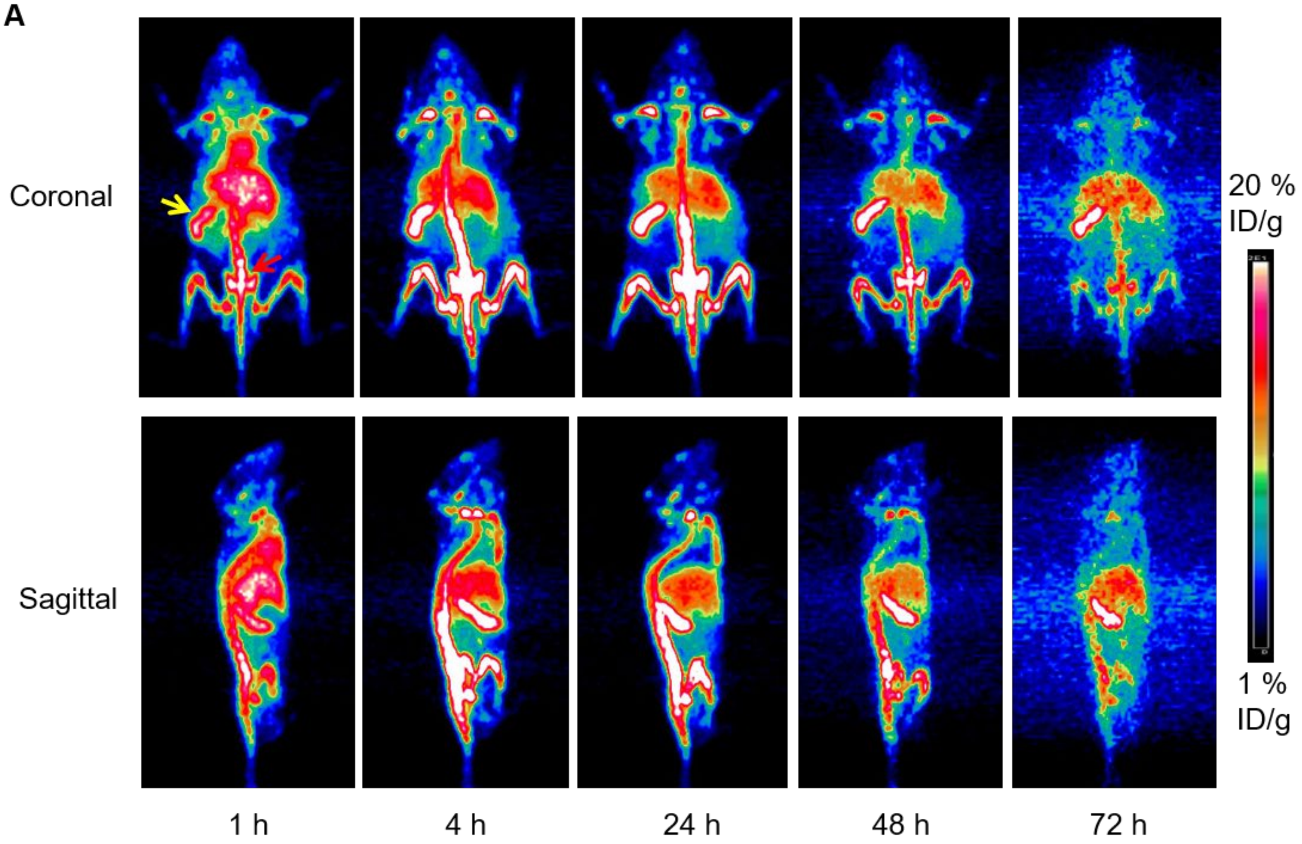
Coronal
Sagittal
20 %
ID/g
1 %
ID/g
1 h
4 h
24 h
48 h
72 h

B

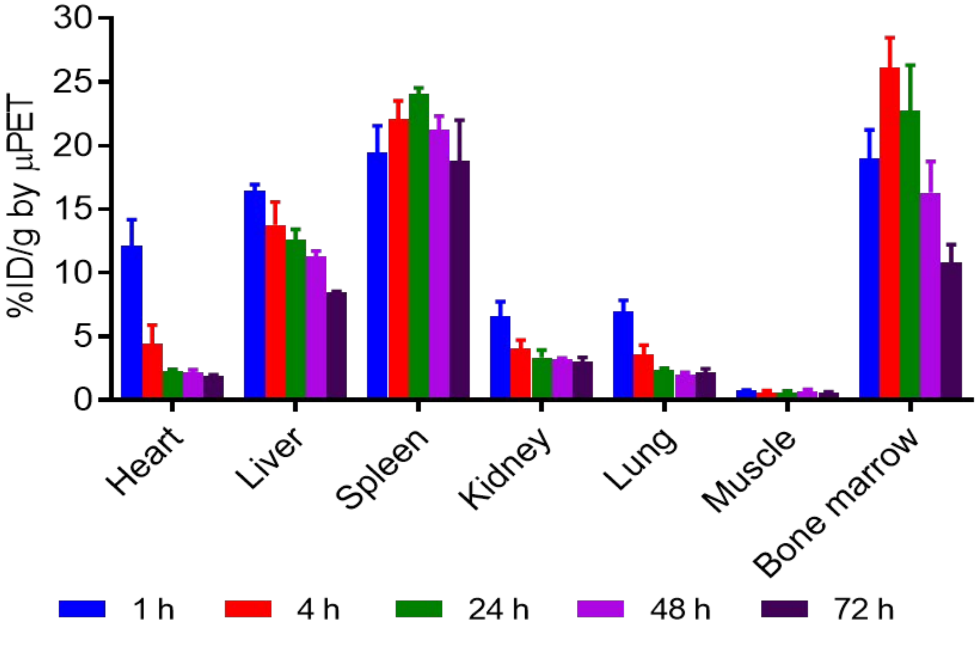
%ID/g by μPET
30
25
20
15
10
5
0
Heart
Liver
Spleen
Kidney
Lung
Muscle
Bone marrow
1 h
4 h
24 h
48 h
72 h

Figure S2. µPET of female nude mice with ^{64}Cu-αCD11b. (**A**) Representative µPET images acquired 1, 4, 24, 48, and 72 h after intravenous injection of ^{64}Cu-αCD11b (red arrow: bone marrow; yellow arrow: spleen). (**B**) Quantitative analysis of organ distribution of ^{64}Cu-αCD11b from images acquired at different time points after radiotracer injection. Data are expressed as mean ± standard deviation (n = 3/group).

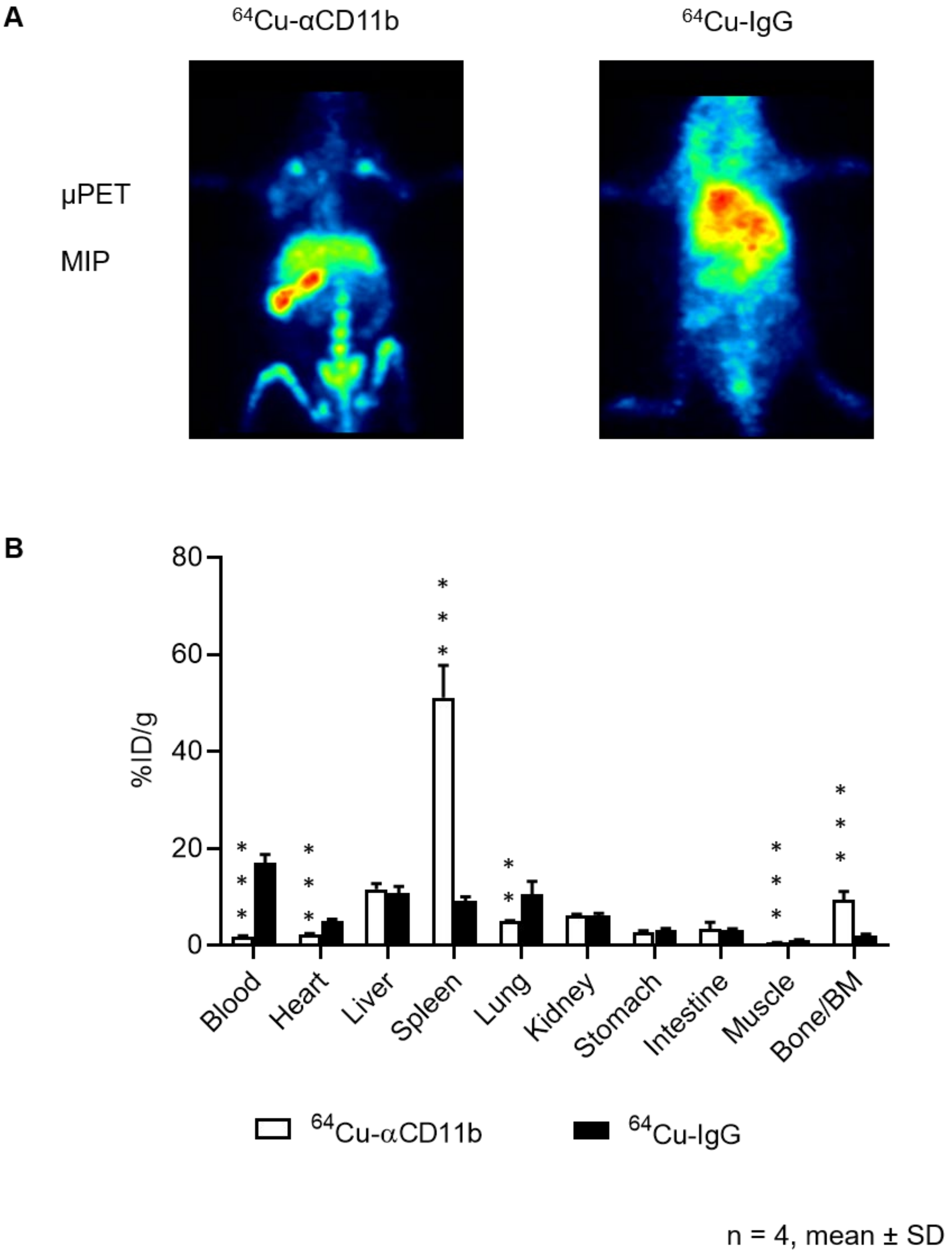

Figure S3. μPET and biodistribution of ^{64}Cu-αCD11b or ^{64}Cu-IgG in normal female 129×1/svJ mice. (**A**) Representative μPET/CT images were acquired 24 h after intravenous injection of ^{64}Cu-αCD11b or ^{64}Cu-IgG. MIP: maximum intensity projection. (**B**) Biodistribution data of ^{64}Cu-IgG control

antibody were compared to ^{64}Cu-αCD11b in mice 24 h after intravenous injection. Data are expressed as mean ± SD (n = 3/group). **, $p < 0.01$; ***, $p < 0.001$.

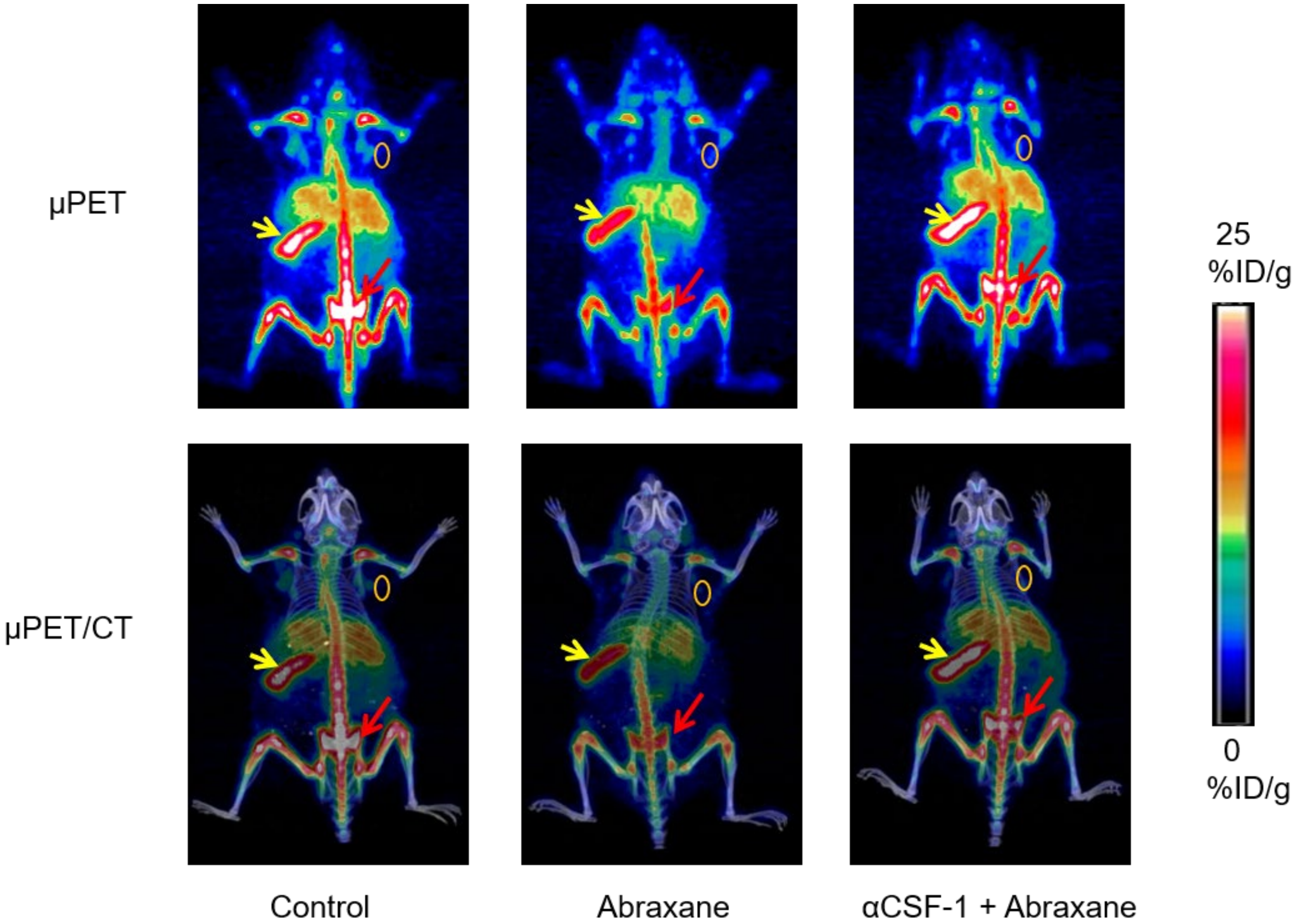

Figure S4. ^{64}Cu-αCD11b μPET-CT of MDA-MB-435 tumor-bearing nude mice treated after a single dose of Abraxane in low scale bar. Representative μPET-CT images (scale bar 0 – 25 %ID/g) acquired 24 h after intravenous injection of ^{64}Cu-αCD11b. Red arrows: bone marrow; yellow arrows: spleen; gold circles: tumor.

A

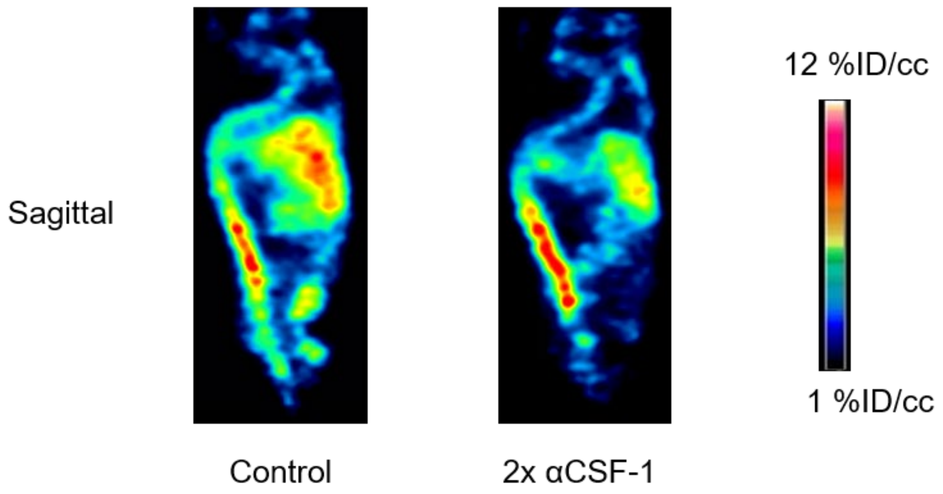

B

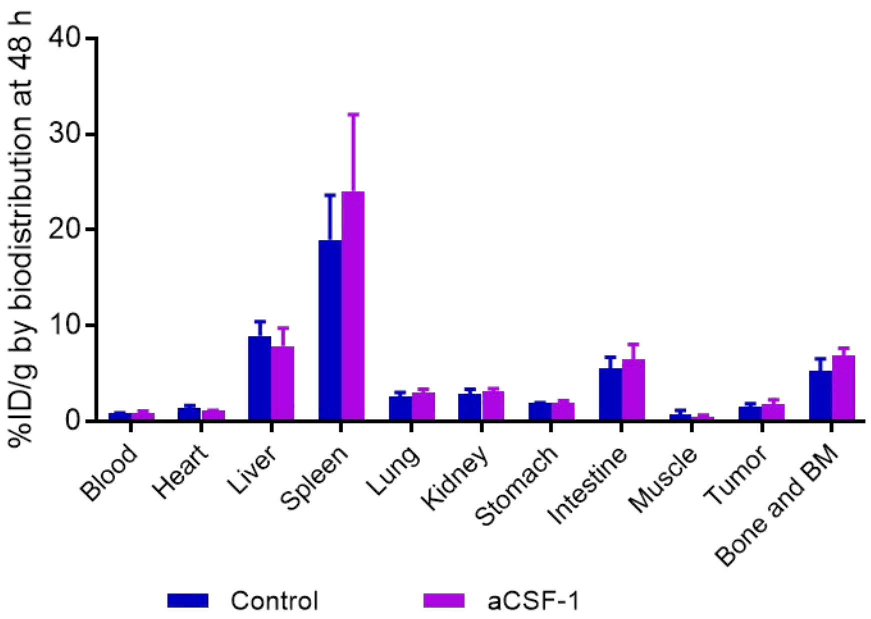

Figure S5. ^{64}Cu-αCD11b μPET and biodistribution of female nude mice without or with αCSF-1 treatment. (**A**) Representative μPET images acquired at 24 h after intravenous injection of ^{64}Cu-αCD11b. (**B**) Biodistribution of ^{64}Cu-αCD11b obtained at 48 h after radiotracer injection. Data are expressed as mean ± standard deviation (n = 3/group).

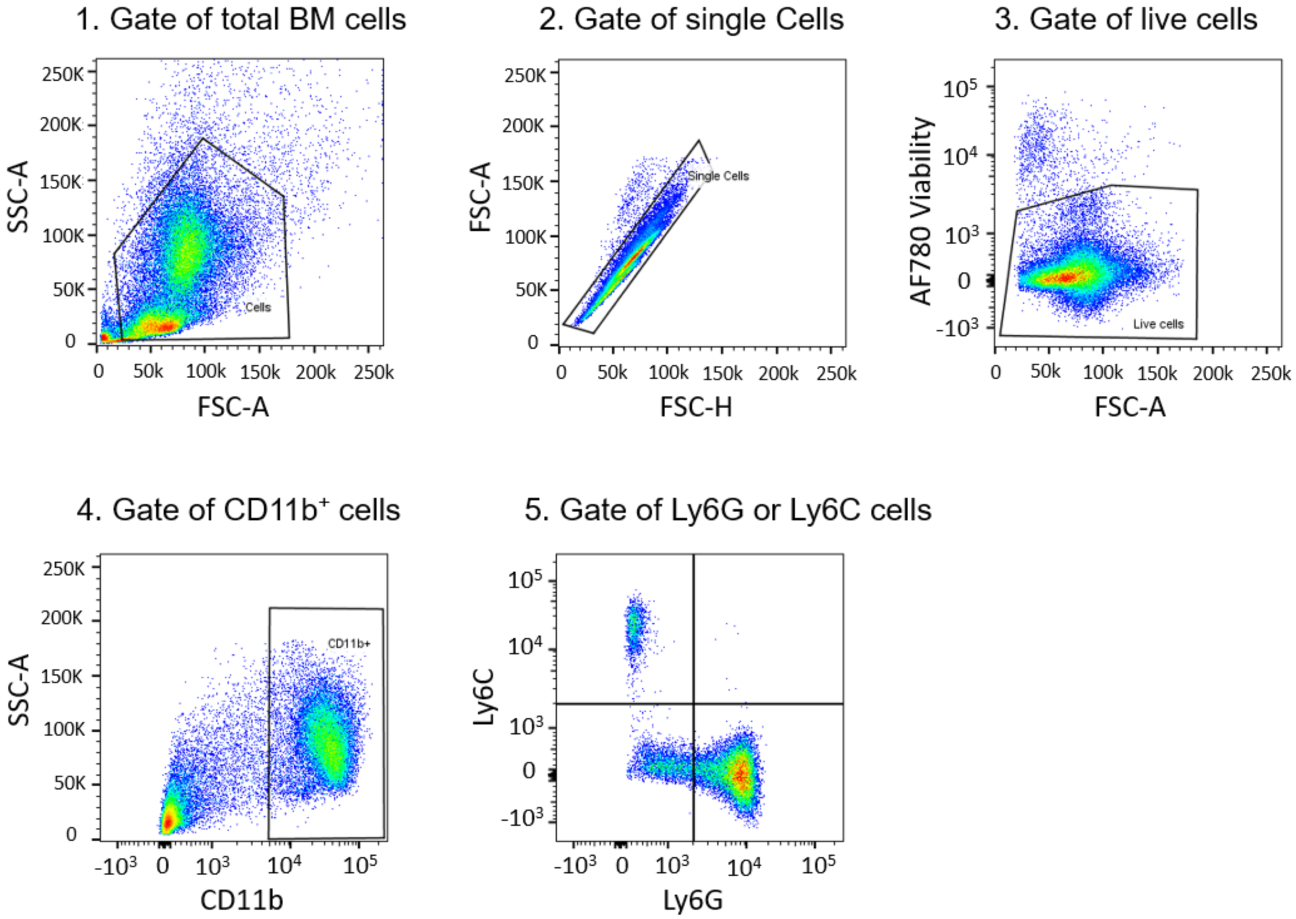

Figure S6. Gating strategy for flow cytometry analysis of bone marrow cell populations. Bone marrow cells were subgated to the level of $CD11b^+$ myeloid cells, then $CD11b^+$ myeloid cells were subgated to $Ly6G^+$ granulocytes or $Ly6C^+$ monocytes.

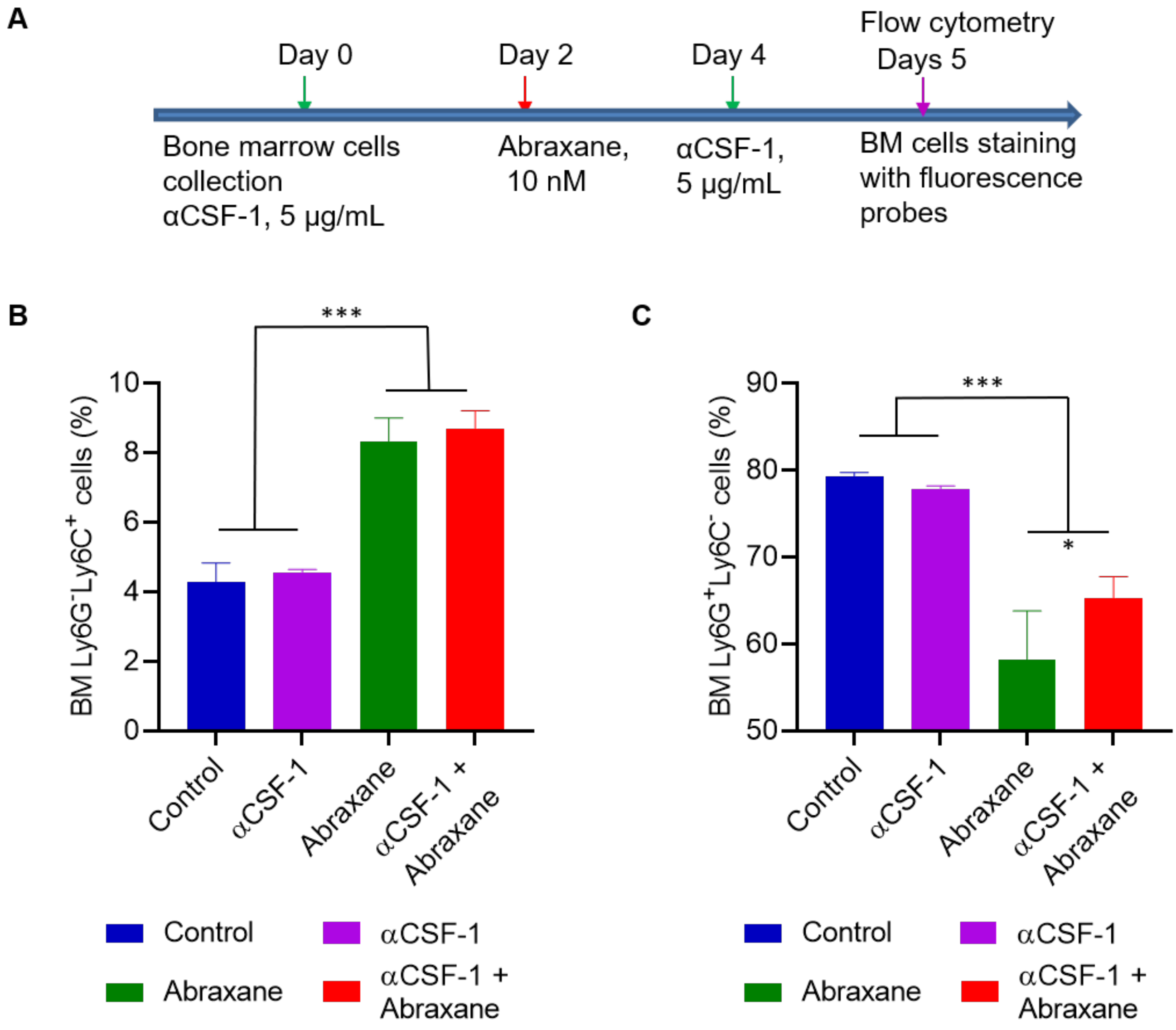

Figure S7. Flow cytometry analysis of bone marrow cells treated with αCSF-1 and/or Abraxane *in vitro*. (**A**) Scheme of experimental design. Bone marrow cells (1×10^6 cells/mL, 2 mL) were treated with αCSF-1 (5 μg/mL) on days 0 and 4 and/or Abraxane (10 nM) on day 2. Bone marrow cells were analysis with flow cytometry on day 5. (**B**) Quantification of Ly6G^{low}Ly6C^{+} monocytic myeloid cells as a percentage of all bone marrow cells. (**C**) Quantification of Ly6G^{+}Ly6C^{low} granulocytic myeloid cells as a percentage of all bone marrow cells. Data in both panels are presented as mean ± SD (n = 3). *, $p < 0.05$; ***, $p < 0.001$.

Reference

1. Cai W, Wu Y, Chen K, Cao Q, Tice DA, Chen X. *In vitro* and *in vivo* characterization of 64Cu-labeled Abegrin, a humanized monoclonal antibody against integrin alpha v beta 3. Cancer Res. 2006; 66: 9673-81.

2. Meares CF, McCall MJ, Reardan DT, Goodwin DA, Diamanti CI, McTigue M. Conjugation of antibodies with bifunctional chelating agents: isothiocyanate and bromoacetamide reagents, methods of analysis, and subsequent addition of metal ions. Anal Biochem. 1984; 142: 68-78.
